# Reply to Charrel, R.N.; Depaquit, J. Comment on “Xu et al. Isolation and Identification of a Novel Phlebovirus, Hedi Virus, from Sandflies Collected in China. *Viruses* 2021, *13*, 772”

**DOI:** 10.3390/v13122422

**Published:** 2021-12-03

**Authors:** Ziqian Xu, Na Fan, Xin Hou, Jing Wang, Shihong Fu, Jingdong Song, Mang Shi, Guodong Liang

**Affiliations:** 1 State Key Laboratory of Infectious Disease Prevention and Control, National Institute for Viral Disease Control and Prevention, Chinese Center for Disease Control and Prevention, Beijing 100052, China; xuzq@ivdc.chinacdc.cn (Z.X.); fanna0925@163.com (N.F.); shihongfu@hotmail.com (S.F.); sjdccdc@163.com (J.S.); 2 The Center for Infection and Immunity Study, School of Medicine, Sun Yat-sen University, Guangzhou 510006, China; houx5@mail2.sysu.edu.cn (X.H.); wangj796@mail2.sysu.edu.cn (J.W.)

Dear Professor Remi N. Charrel and Professor Jerome Depaquit, we thank you for your interest in our research and for your kind suggestions. We agree that the suggested approach for host identification, namely, PCR + NGS (or amplicon-based NGS), which reveals detailed taxonomic compositions from a pooled sample, is more powerful and informative in comparison to the one we had used (i.e., PCR + Sanger sequencing). Despite that, we carried out further analyses (described below), which confirmed that the species identification here is reliable, such that *Phlebotomus chinensis* is the most probable vector for *Heidi virus* (HEDV).

On the one hand, we carefully examined the DNA sequence trace chromatogram obtained from the Sanger sequencing performed in our study, which showed clear and unambiguous signals throughout except for a single position (position 457), which had a mixture of “A” and “T” (Figure 1). Since multiple mixed chromatography trace signals are expected with the presence of more than two species of sandflies, because they shared less than 90% nucleotide identity in the COI gene, we are highly confident to suggest that the majority, if not all, sandflies in the sample belonged to *Ph. chinensis*.

On the other hand, we performed taxonomy profiling of a (different) pooled sample containing HEDV, which was analyzed based on meta-transcriptomic NGS analysis (SRA accession: SRR16970469). *Ph. chinensis* samples were captured in Jul 2020 at Yuncheng of Shanxi Province. The sample collection was performed with light traps placed near a livestock shed that housed dogs and chickens. Species identification was carried out by an experienced field biologist, and subsequently, *Ph. chinensis* samples (n = 50) were pooled for total RNA sequencing. Although the sample characterized here was not the same as the one used in our previous study (none were left after the initial virus isolation procedures), it contained HEDV whose abundance level reached 56.4 Reads per million, or RPM, based on read mapping using bowtie 2 program version 2.3.5 [1], suggesting a relatively high viral load within the sample. Reads from meta-transcriptomics sequencing were then *de novo* assembled using megahit program version 1.2.8 [2] and compared against COI sequence databases using the BLAST program, which revealed three COI-related contigs associated with *Ph. chinensis* (1444.5 RPM), *Bradysia* spp. (8.6 RPM), and an unidentified member of the Order *Diptera* (45.1 RPM), respectively. Given the high abundance level of the HEDV, it is more likely to be associated with *Ph. chinensis*, the dominant species, than the other two species. Furthermore, an analysis of minor variants using Geneious software package version 11.04 [3], based on reads mapped to COI genes, did not suggest the presence of a second species within the sample. Although polymorphism was identified in 24 positions (Table 1), it is too low to imply the presence of another species. Collectively, *Ph. chinensis* is the dominant species within the HEDV-positive samples and the only sandfly species identified here.

Nevertheless, the meta-transcriptomics result here did indicate that the species composition of a pooled sample can be complex, as correctly pointed out by you. To resolve this issue, it requires species composition analyses from more HEDV positive samples, such that a more definite vector–virus relationship can be established. We are currently screening more sandfly pools with meta-transcriptomics as well as PCR + NGS approaches, and hopefully, this matter can be clarified in the future.

## Figures and Tables

**Figure 1:**
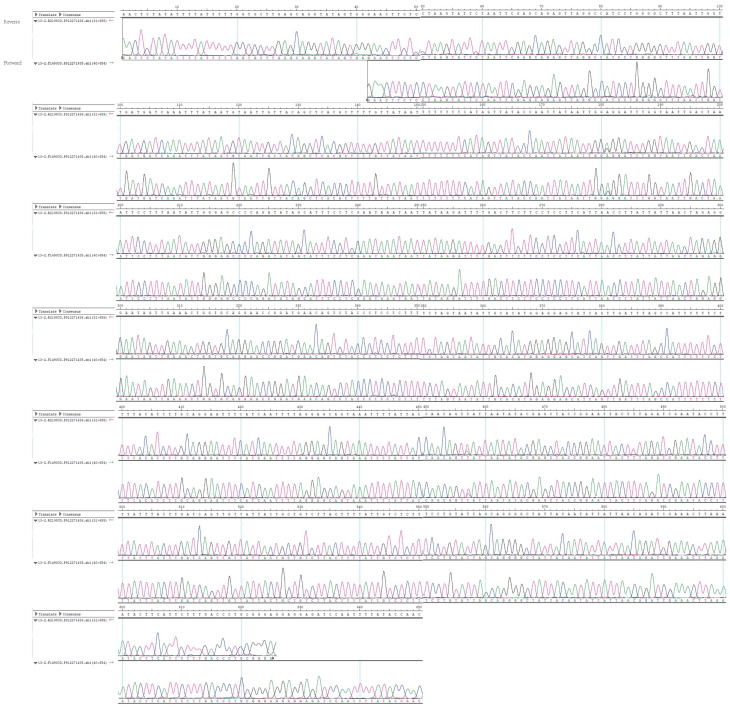
The trace chromatogram for Sanger sequencing of cytochrome c oxidase I (COI) gene within the HEDV positive sample. The amplicon is around 650 nucleotides in length. For each position, both forward and reverse base callings and chromatograms are presented, and their positions and consensus sequences are shown above the chromatograms.

**Table 1:** Minor nucleotide variants of *Ph. chinensis* COI gene in the sample containing HEDV.

Name	Position	Length	Change	Coverage	Polymorphism Type	Variant Frequency
C	1450	1	A -> C	3613	SNP (transversion)	7.70%
C	1299	1	T -> C	2515	SNP (transition)	12.20%
T	1219	1	C -> T	2198	SNP (transition)	13.80%
T	1215	1	C -> T	2193	SNP (transition)	11.40%
C	1152	1	T -> C	4251	SNP (transition)	13.30%
A	1134	1	G -> A	4705	SNP (transition)	18.50%
C	1107	1	T -> C	4541	SNP (transition)	10.30%
A	1023	1	G -> A	6631	SNP (transition)	27.80%
G	966	1	A -> G	6469	SNP (transition)	25.80%
C	831	1	T -> C	5120	SNP (transition)	15.30%
G	783	1	A -> G	5285	SNP (transition)	6.50%
T	675	1	C -> T	6392	SNP (transition)	24.40%
A	571	1	G -> A	10,628	SNP (transition)	9.00%
C	417	1	A -> C	5624	SNP (transversion)	15.60%
T	381	1	C -> T	4386	SNP (transition)	14.00%
T	366	1	C -> T	8409	SNP (transition)	6.90%
A	351	1	G -> A	8213	SNP (transition)	10.60%
G	255	1	A -> G	11,297	SNP (transition)	5.90%
G	129	1	A -> G	7800	SNP (transition)	7.80%
A	114	1	G -> A	6668	SNP (transition)	8.20%
T	96	1	A -> T	5738	SNP (transversion)	11.30%
C	91	1	T -> C	5643	SNP (transition)	8.60%
T	90	1	C -> T	5626	SNP (transition)	20.80%

## Data Availability

All of the materials and data that were used or generated in this study are described and available in the manuscript.
